# Association of Disinhibited Eating and Trait of Impulsivity With Insula and Amygdala Responses to Palatable Liquid Consumption

**DOI:** 10.3389/fnsys.2021.647143

**Published:** 2021-05-03

**Authors:** Yuko Nakamura, Shinsuke Koike

**Affiliations:** ^1^UTokyo Center for Integrative Science of Human Behavior, The University of Tokyo, Tokyo, Japan; ^2^International Research Center for Neurointelligence, The University of Tokyo Institutes for Advanced Study, Tokyo, Japan; ^3^UTokyo Institute for Diversity and Adaptation of Human Mind, The University of Tokyo, Tokyo, Japan; ^4^Center for Evolutionary Cognitive Sciences, Graduate School of Arts and Sciences, The University of Tokyo, Tokyo, Japan

**Keywords:** disinhibited eating, impulsivity, adolescents, food consumption, functional magnetic resonance imaging – fMRI

## Abstract

Eating behavior is not only influenced by the current energy balance, but also by the behavioral characteristics of eating. One of the recognized eating behavior constructs is ‘disinhibited eating,’ which refers to the tendency to overeat in response to negative emotional states or the presence of highly palatable foods. Food-related disinhibition is involved in binge eating, weight gain, and obesity and is also associated with the trait of impulsivity, which in turn, is linked to weight gain or maladaptive eating. However, the relationships among food-related disinhibition, the trait of impulsivity, and the neural substrates of eating behaviors in adolescence remain unclear. Therefore, we designed a functional magnetic resonance imaging (fMRI) study to examine the associations between brain responses to palatable liquid consumption and disinhibited eating behavior or impulsivity in healthy adolescents. Thirty-four adolescents (mean age ± standard deviation = 17.12 ± 1.91 years, age range = 14–19 years, boys = 15, girls = 19) participated in this study. Disinhibited eating was assessed with the disinhibition subscale of the Three-Factor Eating Questionnaire, while impulsivity was assessed using the Barratt impulsiveness scale. Participants received two fMRI sessions−a palatable liquid consumption fMRI and a resting-state fMRI. The fMRI experiment showed that increased disinhibited eating was positively associated with a greater insular response to palatable liquid consumption, while increased impulsivity was positively correlated with a greater amygdala response. The resting-state fMRI experiment showed that increased disinhibited eating was positively correlated with strengthened intrinsic functional connectivity between the insula and the amygdala, adjusting for sex (estimates of the beta coefficients = 0.146, standard error = 0.068, *p* = 0.040). Given that the amygdala and insular cortex are structurally and functionally connected and involved in trait impulsivity and ingestive behavior, our findings suggest that increased disinhibited eating would be associated with impulsivity via strengthened intrinsic functional connectivity between the insula and amygdala and linked to maladaptive eating.

## Introduction

Over the last 40 years there has been a greater than 10-fold increase in the number of school-age children and adolescents with obesity (from 11 million to 124 million; 2016 estimates) (NCD Risk Factor Collaboration (NCD-RisC), 2017). Childhood obesity is one of the most serious global public health problems worldwide. Compared with children with a healthy weight, overweight or obese children are more likely to experience negative consequences (Lobstein et al., 2004), including poorer health in childhood and adulthood, poor academic performance, and a lower chance of being employed as an adult (OECD., 2019). Therefore, understanding the neural substrates of eating behavior or maladaptive eating behavior in children is important to obtain insights for treatment of childhood obesity.

Eating behavior is influenced by the behavioral characteristics of eating. One of the recognized eating behavior constructs is ‘disinhibited eating,’ which refers to the dysregulation toward overeating in the presence of palatable foods or with a negative affect (Stunkard and Messick, 1985). Food-related disinhibition is associated with greater adiposity, over eating, compulsive eating, and binge eating in adults (Crow et al., 2001; Macht et al., 2003; Bellisle et al., 2004; Chaput et al., 2009, 2010; Harden et al., 2009; Houben et al., 2012; Carr et al., 2014; Kruger et al., 2016; Robinson et al., 2016; Blumfield et al., 2018) and children (Bonny et al., 2004; Markowitz et al., 2009; Gallant et al., 2010; Bishop-Gilyard et al., 2011; Rutters et al., 2011; Mailloux et al., 2014; Hootman et al., 2018; Lawless et al., 2020). Food-related disinhibition is also linked to an increased trait of impulsivity (Goldstein et al., 2014; Legenbauer et al., 2018). Impulsivity is a component of the personality concept that involves a tendency to display behavior characterized by little or no forethought, reflection, or consideration of the consequences (Deyoung, 2010). Increased impulsivity contributes to obesity, binge eating, and overeating in adults (McElroy et al., 2005; Cortese et al., 2007; Kaye, 2008; Emery and Levine, 2017) and children (Cortese et al., 2008; Kalarchian and Marcus, 2012; Fields et al., 2013; Schag et al., 2013; Liang et al., 2014; Pearson et al., 2014; Quesada et al., 2018; Kemps et al., 2020).

Neuroimaging studies in adults have shown that food-related disinhibition is associated with increased ventral striatum volumes (Abdo et al., 2020) and decreased middle frontal gyrus gray matter volumes (Yao et al., 2016). Food-related disinhibition is also associated with increased intrinsic resting-state functional connectivity (RSFC) in the frontal motivational system (i.e., orbitofrontal cortex), the premotor cortex such as the pre-/post-central gyrus (Zhao et al., 2017), and the frontoparietal network (Park et al., 2016). Furthermore, food-related disinhibition is associated with the food cue-related functional connectivity between the amygdala and the dorsomedial prefrontal cortex (Dietrich et al., 2016) and increased brain response to food images in the postcentral gyrus (Aviram-Friedman et al., 2018) and supramarginal gyrus (Drummen et al., 2019). In children, lower orbitofrontal cortex volume is observed in participants with increased food-related disinhibition and lower cognitive test performance (Maayan et al., 2011). Food-related disinhibition is also associated with neural activation in response to ingestion of palatable foods in regions related to self-regulation, such as the cuneus and the inferior frontal gyrus (Goldschmidt et al., 2018). Altogether, disinhibition is connected to motivational and reward regions (e.g., the striatum and the prefrontal cortex) and inhibitory control regions, such as the frontal cortex.

Impulsivity is related to responses to food cues in the reward-related regions, such as the orbitofrontal cortex, striatum, amygdala, pallidum, and midbrain (Beaver et al., 2006; Lowe et al., 2009). Impulsivity is also associated with increased activation during anticipation of primary taste reward in the anterior cingulate cortex and amygdala, while RSFC between these regions was negatively correlated with trait impulsivity (Kerr et al., 2015). Furthermore, impulsivity is correlated with increased caudate activation during palatable liquid consumption (Babbs et al., 2013), while impulsivity is correlated with food choice-related brain activation in the striatum (van der Laan et al., 2016). In both adults and children, binge-eating behavior, which is related to impulsivity, is associated with front-striatal functions (Berner and Marsh, 2014). Finally, in children, the difference in the strength of functional connectivity between frontal pole − nucleus accumbens connectivity and inferior parietal lobe − nucleus accumbens connectivity is associated with increased impulsivity, body mass index (BMI), and food approach (Chodkowski et al., 2016). Together, these findings suggest that impulsivity is related to food reward regions including the orbitofrontal cortex, striatum, amygdala, and anterior cingulate cortex.

During the adolescent period, brain regions related to reward, impulsivity, and inhibitory control are dramatically modified (Spear, 2013; Smith et al., 2014; Dai and Scherf, 2019), while adolescents are sensitive to reward stimuli (Kray et al., 2018). Therefore, they are prone to overeating in response to palatable food stimuli (Reichelt, 2016; Reichelt and Rank, 2017). Thus, it is important to examine the relationships between food-related disinhibition, impulsivity, and the neural circuits of eating behavior in children. Given that food-related disinhibition and impulsivity are related to each other and that brain regions related to each behavioral characteristic overlap in the eating behavior regions (e.g., the striatum and the amygdala), these behavioral characteristics may jointly modulate eating behavior. However, little is known about the associations between the brain response to food consumption, impulsivity, and food-related disinhibition in children. Furthermore, although intrinsic RSFC in the reward and motivational regions was associated with food-related disinhibition or impulsivity (Kerr et al., 2015; Park et al., 2016; Zhao et al., 2017), little is known about the associations among RSFC, food-related disinhibition, and impulsivity.

Since food-related disinhibition and impulsivity are related to each other and are also involved in a similar brain network for ingestive behavior, we assumed that these behavioral traits would modulate eating behavior in a coordinated manner. To address this hypothesis, we designed a functional magnetic resonance imaging (fMRI) study in adolescents. We focused on the insular and amygdala responses to palatable liquid consumption. The primary gustatory area is located in the insular cortex (Chikazoe et al., 2019), while the insular responses to food cues are associated with greater adiposity (Rothemund et al., 2007), binge-eating behavior (Schienle et al., 2009), and food reward (van Rijn et al., 2018). Furthermore, functional connectivity in the insula is associated with impulsivity (Wisner et al., 2013; Chen et al., 2016; Zheng et al., 2019; Kim et al., 2021) insular activation related to reward processing has been linked to impulsivity (Villafuerte et al., 2012) and increased response to food orders in the insula is connected to greater impulsivity in adolescents (de Celis-Alonso et al., 2019). Moreover, behavioral disinhibition is associated with hypometabolism (Schroeter et al., 2011), traumatic injuries (Knutson et al., 2015), and tissue loss (Rosen et al., 2005) in the insula. In addition, disinhibited eating has been associated with increased regional blood flow in the insula after liquid-meal consumption (DelParigi et al., 2005). The amygdala is also reactive to gustatory stimuli (Doornweerd et al., 2018; Nakamura et al., 2020) and its response is associated with increased BMI (Stoeckel et al., 2008; Sun et al., 2015), binge-eating (Schienle et al., 2009), and impulsivity (Kim et al., 2018). Trait impulsivity is associated with increased amygdala activation during anticipation of a rewarding taste, and intrinsic functional connectivity in the amygdala is negatively correlated with trait impulsivity (Kerr et al., 2015). Moreover, trait disinhibition is associated with reduced amygdala volume (Vieira et al., 2015) and amygdala response to affective visual stimuli (Foell et al., 2016). Furthermore, disinhibited eating is correlated with reduced strength of functional connectivity in the amygdala (Dietrich et al., 2016). In addition, the amygdala and insula are structurally and functionally connected (Ghaziri et al., 2018; Sylvester et al., 2020). Given that the amygdala and insula play critical roles in ingestive behavior, food-related disinhibition, and impulsivity, we set these as regions of interest (ROIs) to test the associations between brain responses to palatable liquid consumption and food-related disinhibition or impulsivity. Disinhibited eating behavior was measured using the disinhibition subscale of the 51-item Three-Factor Eating Questionnaire (TFEQ) (Stunkard and Messick, 1985; Adachi et al., 1992). The TFEQ is a self-report questionnaire designed for adolescents and adults (ages 12 and up) to assess three factors of eating behavior: cognitive restraint of eating, disinhibition, and hunger. Disinhibition has been shown to be a critical predictor of BMI, weight gain, and obesity (Bryant et al., 2008, 2019; Lesdéma et al., 2012). Impulsivity was measured using the Barratt Impulsiveness Scale Version 11 (BIS-11), which was designed to assess the personality/behavioral construct of impulsiveness. The BIS-11 assesses a single general factor (reflecting impulsivity) and three subdomains: attentional impulsiveness (inability to focus attention or concentrate), motor impulsiveness (acting without thinking), and non-planning impulsiveness (lack of future orientation or forethought). This self-report questionnaire has been adapted to assess impulsivity in adolescents (Pechorro et al., 2016; Huang et al., 2017). Impulsivity has been linked to obesity (Bénard et al., 2017), poor diet quality (Bénard et al., 2019), and eating disorders, such as binge-eating (Meule, 2013). Furthermore, we tested whether the insular − amygdala RSFC measured by a resting-state fMRI (rs-fMRI) experiment was explained by food-related disinhibition or impulsivity. We hypothesized that an increased amygdala or insular response to palatable liquid consumption would be associated with increased food-related disinhibition or impulsivity, while the increased intrinsic insular − amygdala RSFC would be related to increased food-related disinhibition or impulsivity.

## Materials and Methods

### Participants

Thirty-four healthy adolescents (mean age ± standard deviation = 17.11 ± 1.9 years, age range = 14–19 years, *N* = 15 boys, *N* = 19 girls) participated in the current study (Table 1). Participants were recruited from the metropolitan area of Tokyo. All participants also participated in another study, which aimed to construct an MRI open database using a standardized MRI sequence. Details of this study have been described elsewhere (Koike et al., 2021). All participants and parents/guardians of the high school or middle school participants provided written informed consent and the study was approved by the Ethics Committee of the Department of Arts and Sciences, The University of Tokyo (Approval No. 513–2 and 20-297). All participants were teenagers and free of current or prior psychiatric or neurological disorders, chronic and acute physical illnesses including diabetes, current psychopharmacological medication, eating disorders, current dieting behavior, alcoholism, use of tobacco or drugs, history of head injury with loss of consciousness, chemosensory impairments, and food allergies. Individuals were included only if they were comfortable with being inside an MRI scanner.

**TABLE 1:** Demographics.

	Age (years)	Gender (boys/girls)	BMI	Disinhibition	Attentional	Motor	Non-planning	Total
Mean	17.12	15/19	20.78	6.71	26.15	32.35	35.27	93.64
*SD*	1.92		2.05	3.21	5.58	7.31	7.09	16.84

*SD, standard deviation; BMI, body mass index.*

### Experimental Overview

**FIGURE 1:**
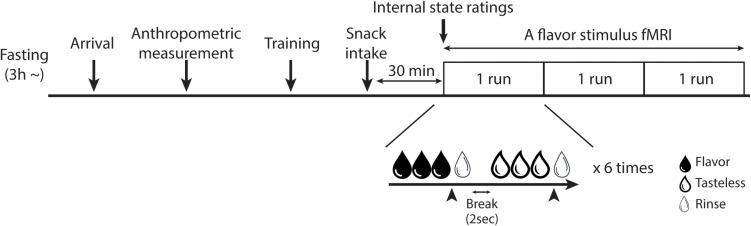
Overview of the fMRI experiment. The training session was only set for the flavor stimulus fMRI experiment. In the flavor stimulus fMRI experiment, the participant performed three runs of the flavor stimulus fMRI scan, thus, 18 trials were performed for each solution in total. The participant was instructed to stop receiving solutions whenever they would have liked to stop it. Arrowheads indicate the timing of the participant to press the button to stop the drinking solutions. In the rs-fMRI experiment, the participant performed one run of the rs-fMRI scan.

All participants underwent two fMRI scans: a flavor stimulus fMRI scan and an rs-fMRI scan. Since participants underwent the rs-fMRI scan as a part of another study (Koike et al., 2021), each fMRI scan was performed on different days. The order of the scans was randomized for each participant and each scan was performed within a month. The participants were instructed to abstain from any food or drinks, except for water, for at least 3 h before their visit to our laboratory. After their arrival, anthropometric measurement was performed on the day of one of the fMRI scans and BMI was calculated. At 30 min before the fMRI scan, participants were instructed to consume pre-fixed snacks (280 kcal) to standardize their internal states (e.g., hunger and fullness). The percentage of calories of the pre-fixed snack in the total estimated daily calories needed to maintain energy balance was calculated based on the Japanese dietary reference intake published by the Ministry of Health, Labor and Welfare in 2020. For boys, calories of the pre-fixed snack were 10.00 – 10.76% of the total estimated daily calories needed and, for girls, it was 11.67 – 12.17%. Participants were then escorted to the fMRI scanner room to undergo the fMRI scan (Figure 1). By the end of the second fMRI scan, disinhibited eating behavior and the trait of impulsivity were measured by the subscales of the Japanese version TFEQ (Stunkard and Messick, 1985; Adachi et al., 1992) and the BIS-11 (Patton et al., 1995; Someya et al., 2001), respectively.

### Disinhibited Eating Behavior and Trait of Impulsivity

Disinhibited eating behavior was measured using the disinhibition subscale of the 51-item TFEQ. The disinhibition subscale consists of 16 items and its score is a sum of the item scores of 0 or 1 (score range = 0–16). Higher scores denote higher levels of disinhibited eating.

The trait of impulsivity was measured by the BIS-11. The BIS-11 contains a total of 30 items scored on a Likert scale (ranging from never = 1 point to very frequently = 6 points), which yields impulsivity measures on three scales: attentional (inability to focus or concentrate), motor (tendency to act without thinking), and non-planning impulsivity (lack of future planning and forethought). The total score ranges from 30 to 180, with a higher score indicating greater impulsivity.

### A Training Session for the Flavor Stimulus fMRI Scan

For the flavor stimulus fMRI scan, all participants underwent an entire run of the flavor stimulus fMRI task for training. For this fMRI scan, we delivered a commercially available popular beverage. Given that disinhibited eating would be associated with food reward (Stunkard and Messick, 1985), to measure the brain response to rewarding stimuli, all participants were shown pictures of five beverages in the product packages at enrollment, and instructed to select the most preferred beverage from five options (orange juice, lemonade, strawberry milk, sport drink, and yogurt flavored drink) as the flavored solution. They were also instructed not to select the option they had previously not experienced. Each beverage was adjusted to a calorie level of 40 kcal/100 mL. We did not notify participants that the calorie level of the flavored solution was adjusted, and no participant noticed it. A tasteless solution was used as a control. Participants were presented with two tasteless solutions−original artificial saliva (25 mM KCl + 2.5 mM NaHCO_3_) (O’Doherty et al., 2001) and a twofold diluted original artificial saliva−and were instructed to select the one that was most tasteless as the control solution.

The flavored and tasteless solutions were randomly delivered into the participant’s mouth via a homemade gustometer and a tailor-made mouthpiece. This homemade gustometer was adopted from our previous studies (Nakamura et al., 2011, 2013, 2020). It was placed in the MRI control room, and an assembly of the following four parts: solution bottles, solenoid valves, flow meters, and a personal computer to control the solenoid valves. Two solution bottles, set at 175 cm height, dripped liquid through Teflon tubes to deliver solutions to each participant’s mouth via a mouth-piece. The flow rate was set at 2.5 mL/min by flow maters, and the timing of delivery of the solutions was regulated with the solenoid valves, which were controlled by a single-board microcontroller (Arduino UNO R3) using software (MATLAB 2016b; The MathWorks, Inc., Natick, MA, United States). To create a mouthpiece, a commercially available night mouthguard was heated and modified to fit the participant’s upper jaw. Then, Teflon tubes for solution delivery were attached right under the front teeth position using hot-melt adhesive. After the mouthpiece was made, the participant wore it to confirm it was comfortable. Participants were instructed to naturally swallow the solutions whenever they wanted to. They were also instructed not to try holding the solutions in their mouth, because if they would swallow a lot liquid at once, their head would move a lot. To measure the brain response to rewarding stimuli and to avoid habituation to the flavored solutions, patients were also instructed to stop the solution delivery when they did not desire to consume it. This was achieved using a button attached to a personal computer that switched off the solenoid valves. The tasteless solution was delivered for 3 s as a rinse fluid following each delivery of the flavored or tasteless solution via the tailor-made mouthpiece. There was a 2-s break between the trials. During flavor or tasteless conditions, each participant was shown an instruction that they could stop receiving a solution whenever they wished. During rinse and break durations, a fixation point was presented on the screen. No visual cues to indicate when or which solutions would be delivered were shown. Each solution was presented six times, with a total of 12 trials performed per one run.

### fMRI Scan Sessions

Immediately before each fMRI scan, all participants were asked to rate their hunger and fullness states (the internal state) using an eight-point Likert scale (1 = ‘not at all’; 8 = ‘more than ever’).

For the flavor stimulus fMRI scan, participants were also instructed to receive the flavored solution and rate their desire to drink the solution (wanting) and how much they liked the solution (liking) after internal state ratings. All participants performed three runs of the flavor stimulus task, with 18 trials for each solution in total. In the flavor fMRI scan, the duration of the flavor stimuli delivery was 13.9 ± 5.53 s (mean ± standard deviation) and that for the tasteless solution was 6.9 ± 3.6 s. The averaged scan time for one run was 9 min 59.5 s ± 2 min 51.6 s.

For the rs-fMRI scan, participants were instructed to relax and lie still in the scanner while remaining calm and awake. All participants underwent one run of the rs-fMRI scan (10 min 10 s). During rs-fMRI scanning, each participant was instructed to fix their gaze on a white cross on a black screen, which was projected onto a mirror attached to the head-coil from the monitor that was set behind the bore of the MRI machine.

### Image Acquisition

MR images were acquired on a 3.0 Tesla MRI scanner (Magnetom Prisma, Siemens Medical Systems, Munich, Germany) using a 64-channel head/neck coil. Anatomical images were acquired using a T1-weighted three-dimensional MPRAGE protocol (repetition time = 1900 ms, echo time = 2.53 ms, flip angle = 9°, field of view = 256 mm × 256 mm, resolution = 1.0 mm × 1.0 mm × 1.0 mm). For the flavor stimulus fMRI scan, T2^∗^-weighted images reflecting blood oxygen level-dependent signals were acquired using gradient-echo echo-planar imaging (repetition time = 2000 ms, echo time = 25 ms, 39 slices, flip angle = 80°, field of view = 192 mm × 192 mm, resolution = 3.0 mm × 3.0 mm × 3.0 mm) in an interleaved manner. For the rs-fMRI scan, echo-planar imaging images were acquired with the following protocol (repetition time = 2500 ms, echo time = 30 ms, 38 slices, flip angle = 80°, field of view = 212 mm × 212 mm, resolution = 3.3 mm × 3.3 mm × 4.0 mm) in ascending order.

### Data Analysis for Disinhibited Eating Behavior and the Trait of Impulsivity

All statistical analyses were performed using R statistical software (v4.0.2^1^; R Foundation for Statistical Computing, Vienna, Austria). The threshold for the following exploratory analyses was set at *p* < 0.05. Given that the effect of maturation on the brain or physical development would be different between boys and girls (De Bellis et al., 2001; Ogden et al., 2012), sex differences in disinhibited eating, impulsivity, or BMI were tested by the two-sample *t*-test.

Correlations between age or BMI and disinhibited eating or impulsivity, and between disinhibited eating and impulsivity, were examined by Pearson’s correlation analysis, as maturation is related to impulsivity and self-regulation, adiposity is associated with disinhibited eating and impulsivity, and impulsivity and disinhibited eating are related to each other.

### fMRI Analysis

#### Flavor Stimulus fMRI Data

All analyses for flavor stimulus fMRI data were performed using SPM 12 (Wellcome Trust Department of Cognitive Neurology, London, United Kingdom) on MATLAB (Release 2016b; The MathWorks, Inc.). Preprocessing was performed for all MRI images as follows: (1) correction for image distortion using field mapping (Jenkinson, 2003), (2) slice timing correction, (3) realignment, (4) normalization, (5) correcting signal drift using detrending software (Macey et al., 2004), and (6) spatial smoothing with a 6-mm full-width at half maximum isotropic Gaussian kernel. After conventional preprocessing, a covariate matrix for motion correction at the participant level analysis was generated using Artifact Detection Tools (Mazaika et al., 2005). The covariate matrix included six movement parameters (three rotations and three translations) and the time at which the image volumes with the *z*-normalized global brain activation exceeded three standard deviations from the mean of the run or showed > 1 mm of movement was considered. The exclusion criterion for head-motion was > 1 voxel size (3.0 mm × 3.0 mm × 3.0 mm); no participants met this exclusion criterion. The mean head-motion values (x, y, z) were 0.19 ± 0.08 mm (range = 0.05–0.55 mm), 0.28 ± 0.17 mm (0.11–1.29 mm), and 0.93 ± 0.53 mm (0.10–2.93 mm), respectively.

A general linear model analysis was performed at the participant level, in which we modeled a boxcar function with a sustained epoch representing each stimulus duration and the boxcar function was convolved using the canonical hemodynamic response function, which was stored in SPM 12 software. The covariate matrix of motion was included in the model as a covariate of no interest. To omit the effect of low-frequency noise, we set a 270-Hz high-pass filter for the flavor stimulus fMRI data. The [flavored solution > tasteless solution] contrast was created for individual participants.

Contrast images of [flavored solution > tasteless solution] from the participant level analysis were entered into the group level analysis. A one-sample *t*-test was performed to test the effect of [flavored solution > tasteless solution]. The predicted effect was tested using an ROI approach. All ROIs were created using the automated anatomical labeling mask (Tzourio-Mazoyer et al., 2002) in the WFU Pickatlas toolbox (Maldjian et al., 2003). We included the following ROIs based on previous reports examining the brain responses to taste or flavored gustatory solutions (van der Laan et al., 2011; Veldhuizen et al., 2011; García-García et al., 2013; Huerta et al., 2014; Pursey et al., 2014; van Meer et al., 2016; Yeung et al., 2017): the insular cortex, thalamus, striatum, midbrain, pons, amygdala, hippocampus, parahippocampus, orbitofrontal cortex, ventromedial prefrontal cortex, anterior cingulate cortex, midcingulate cortex, and pre-/post-central gyrus. The t-map threshold for all image analysis was set at *p*_*uncorrected*_ < 0.001 and a cluster size at a minimum of five contiguous voxels with a peak voxel survived at *p* < 0.05 corrected for multiple comparisons using the family wise error (FWE) rate across an anatomical ROI. The FWE correction method, implanted in the SPM software, was used. We have reported clusters with peak voxels that survived at *p*_*FWE–corrected*_ < 0.003 (0.05/14) as significant. Unpredicted voxels were considered significant at *p* < 0.05 FWE corrected across the entire brain.

To assess the correlations between the individual [flavored solution > tasteless solution] contrast and trait of impulsivity or disinhibited eating behavior, a group-level voxel-wise linear regression analysis with impulsivity or disinhibited eating as a covariate of interest was performed on individual [flavored solution > tasteless solution] contrasts. BMI was included as a potential confounder in the linear regression analysis with impulsivity. For these analyses, we set the amygdala (468 voxels) and insula cortex (3628 voxels) as ROIs based on previous studies (Rothemund et al., 2007; Schienle et al., 2009; Kerr et al., 2015; Sun et al., 2015; van Rijn et al., 2018; Chikazoe et al., 2019; Nakamura et al., 2020) showing that the insula and amygdala were related to taste perception, reward, binge-eating, and impulsivity. We have reported clusters with peak voxels that survived at *p*_*FWE–corrected*_ < 0.025 (0.05/2) as significant. Unpredicted voxels were considered significant at *p* < 0.05, FWE corrected across the entire brain.

#### rs-fMRI Data

All analyses for rs-fMRI data were performed using FSL software (v6.0) (Jenkinson et al., 2012). The first four volumes of each functional time-series were excluded from the analysis to allow for magnetization equilibrium. Preprocessing was conducted as follows: (1) head-motion correction via realignment of the time-series to the middle volume; (2) field map-based distortion correction; (3) slice-timing correction; (4) non-brain tissue removal using the brain extraction tool; (5) spatial smoothing with a 5-mm full-width at half maximum Gaussian kernel; and (6) high-pass temporal filtering (1/150 Hz cutoff). One participant was excluded because of poor co-registration of functional and three-dimensional anatomical data. The exclusion criterion for head-motion was > 1 voxel size (3.3 mm × 3.3 mm × 4.0 mm); no participant met this exclusion criterion. Thus, data from all participants were included in further rs-fMRI analyses. The mean head-motion values (x, y, z) were 0.15 ± 0.09 mm (range = 0.05–0.40 mm), 0.38 ± 0.26 mm (0.14–1.35 mm), and 0.80 ± 0.54 mm (0.19–2.91 mm), respectively. To remove the head-motion effects, we employed ICA-AROMA using a data-driven method to identify and remove motion-related independent components from rs-fMRI data (Pruim et al., 2015a, b). Furthermore, derivative of root mean square variance over voxels were calculated to quantify the mean change in image intensity between time-points (Power et al., 2012). Time-series were then extracted from the white matter and cerebrospinal fluid using preprocessed time-series data. Motion-regressors, derivative of root mean square variance over voxels, and white matter and cerebrospinal fluid time-series were included in a confounder matrix.

To calculate the functional connectivity between the brain regions related to disinhibited eating and impulsivity based on results of the flavor stimulus fMRI data analysis, a seed-based rs-fMRI analysis was performed. To create a connectivity map, the left insular region related to disinhibited eating behavior ([x, y, z] = [−40, 2, −12], size = 13 voxels) was set as a seed based on the results of the flavor stimulus fMRI data analysis and the time-series was extracted from non-smoothed preprocessed rs-fMRI data. The extracted time-series from the seed was included into a general linear model using FSL software (fMRI Expert Analysis Tool). This model included a confounder matrix as a nuisance covariate. From the individual connectivity maps, the connectivity value (*z*-value) was extracted from the left amygdala region related to the trait of impulsivity ([x, y, z] = [−22, 0, −24], cluster size = 8) based on the results of the flavor stimulus fMRI data analysis.

#### Ratings for Internal State and Flavor Stimuli

Hunger or fullness would influence the brain response to a flavored solution or resting-state activity. Therefore, to see if participants were too hungry or too full at one of the fMRI sessions, we tested the effect of internal state (hunger and fullness) and fMRI session (flavor-stimulus fMRI session and rs-fMRI session) on the ratings for internal state using a two-way repeated-measures analysis of variance (ANOVA) with the within-participant factors internal state (hunger and fullness) and experiment (the flavor stimulus fMRI and rs-fMRI). The internal state would also influence participants’ liking or wanting for flavored solutions, which could influence the brain response to palatable flavored solutions. Thus, the associations between internal state ratings (hunger and fullness) and stimulus ratings (liking and wanting) were tested using Pearson’s correlation analysis. For these exploratory analyses, the threshold was set at *p* < 0.05.

### Moderation Effects of Disinhibited Eating or Impulsivity on Associations Between Impulsivity- and Disinhibition-Related Brain Responses to Flavor Solution Consumption

To test whether disinhibited eating and impulsivity would influence the association between brain responses related to disinhibited eating and brain responses related to impulsivity, modulation analysis was performed using the ‘gvlma’ package (v1.0.0.3) in R software (Peña and Slate, 2006). Based on the results of the regression analysis for the flavor-stimulus fMRI data (see section Result “Association of Brain Response With Disinhibited Eating or Impulsivity”), the responses related to disinhibited eating or impulsivity were extracted as beta values from all participants. The response related to disinhibited eating was entered as a dependent variable and the response related to impulsivity and disinhibited eating or impulsivity were entered as explanatory variables into the first step of the regression analysis. In the second step, the interaction term between the response related to impulsivity and disinhibited eating or impulsivity was entered.

### Associations Between Resting State Functional Connectivity and Disinhibited Eating or Impulsivity

The effect of disinhibited eating on functional connectivity between the disinhibited eating-related region and the impulse-related region was tested using a multiple linear regression analysis including sex as a potential confounder. Boys were set as the reference group for sex. The effect of impulsivity on the functional connectivity was also tested using a multiple linear regression analysis including age as a potential confounder.

## Results

### Associations Between Demographics and Disinhibited Eating or Impulsivity

Girls had greater disinhibited eating than boys (*p* = 0.002, *t* = 3.26). There was no significant effect of sex on impulsivity or BMI (*p* > 0.3 for both).

Higher age was positively correlated with higher total, attentional, and motor, but non-planning, impulsivity (*p* = 0.012, *r* = 0.43; *p* = 0.002, *r* = 0.51; *p* = 0.026, *r* = 0.38; and *p* = 0.168, *r* = 0.24, respectively). Age was positively associated with disinhibited eating (*p* = 0.036, *r* = 0.36).

There was a trend toward a positive association of BMI with motor impulsivity (*p* = 0.09, *r* = 0.29).

There was no significant correlation between disinhibited eating and impulsivity (*p* > 0.16).

### Ratings for Internal State and Flavor Stimuli

The two-way repeated ANOVA showed that there was no significant difference in internal state (hunger or fullness) or experiment (flavor-stimulus fMRI and rs-fMRI), or a significant effect of interaction between the internal state and the experiment on internal state ratings [*F*(1,33) = 2.73, *p* = 0.108; *F*(1,33) = 0.087, *p* = 0.770; *F*(1,33) = 2.08, *p* = 0.1.58, respectively]. Therefore, we assumed that participants were not full or hungry at each fMRI scan (Supplementary Table 1).

The ratings for wanting and liking were 5.32 ± 0.94 and 5.91 ± 0.97, respectively. Therefore, we assumed that the solutions were rewarding for all participants.

There was no significant correlation between internal states and ratings for liking and wanting (*p* > 0.27).

### Brain Response to Flavor Stimuli

A brain response to [flavored solution > tasteless solution] was observed in the insula, amygdala, hippocampus, parahippocampus, striatum (putamen), pons, and thalamus (lateral thalamic nuclei) (Supplementary Table 2). After multiple testing correction, brain response in the amygdala, hippocampus, and parahippocampus survived as significant.

There was no significant unpredicted response.

### Association of Brain Response With Disinhibited Eating or Impulsivity

The amygdala response to [flavored solution > tasteless solution] was positively associated with total impulsivity ([x, y, z] = [−22, 0, −24], z = 3.45, *p*_*FWE–corrected*_ = 0.038, cluster size = 5) and motor impulsivity ([x, y, z] = [−22, 0, −24], z = 3.73, *p*_*FWE–corrected*_ = 0.016, cluster size = 8). A *post hoc* statistical power analysis was performed by G^∗^Power software (Faul et al., 2007) to calculate the statistical power of these correlation analyses with beta values from each cluster, and the statistical power with total impulsivity was 0.969 and that of motor impulsivity was 0.980. After multiple testing correction, the association between brain response and total impulsivity did not survive as significant. On adjusting for BMI, the association with total impulsivity ([x, y, z] = [−22, 0, −24], z = 3.33, *p_*FWE–corrected*_* = 0.054, cluster size = 3) or motor impulsivity ([x, y, z] = [−22, 0, −24], z = 3.54, *p_*FWE–corrected*_* = 0.029, cluster size = 5) did not survive as significant.

**FIGURE 2:**
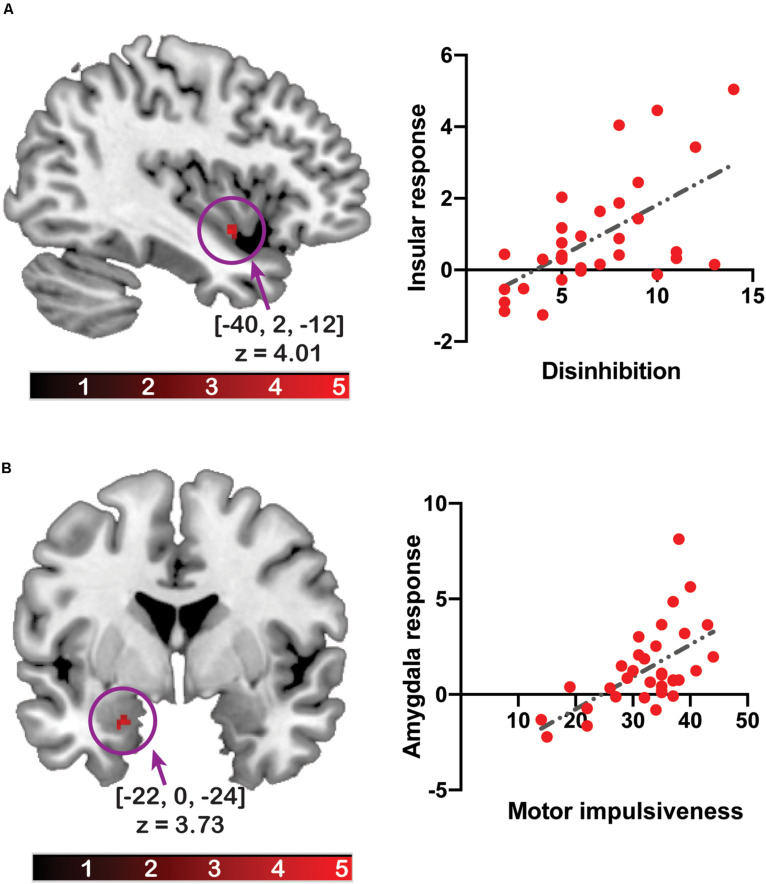
The relationship between the brain response to the flavor stimuli and disinhibited eating or impulsivity. The *y*-axis of the scatter plot depicts beta values extracted from the cluster related to disinhibited eating **(A)** or motor impulsiveness **(B)**. The *x*-axis of the scatter plot depicts disinhibited eating **(A)** or motor impulsiveness **(B)**. The coordinates and *z*-values indicate those of the peak voxels. The colored bars depict the *t*-values.

The insular response was positively associated with disinhibited eating ([x, y, z] = [−40, 2, −12], z = 4.01, *p*_*FWE–corrected*_ = 0.043, cluster size = 13) (Figure 2). The *post hoc* statistical power analysis revealed that the statistical power of this correlation analysis was 0.988. After multiple testing correction, the association between brain response and disinhibited eating did not survive as significant.

There was no significant unpredicted association.

### Moderation Effects of Disinhibited Eating or Impulsivity on Associations Between Impulsivity- and Disinhibited Eating-Related Brain Response

**FIGURE 3:**
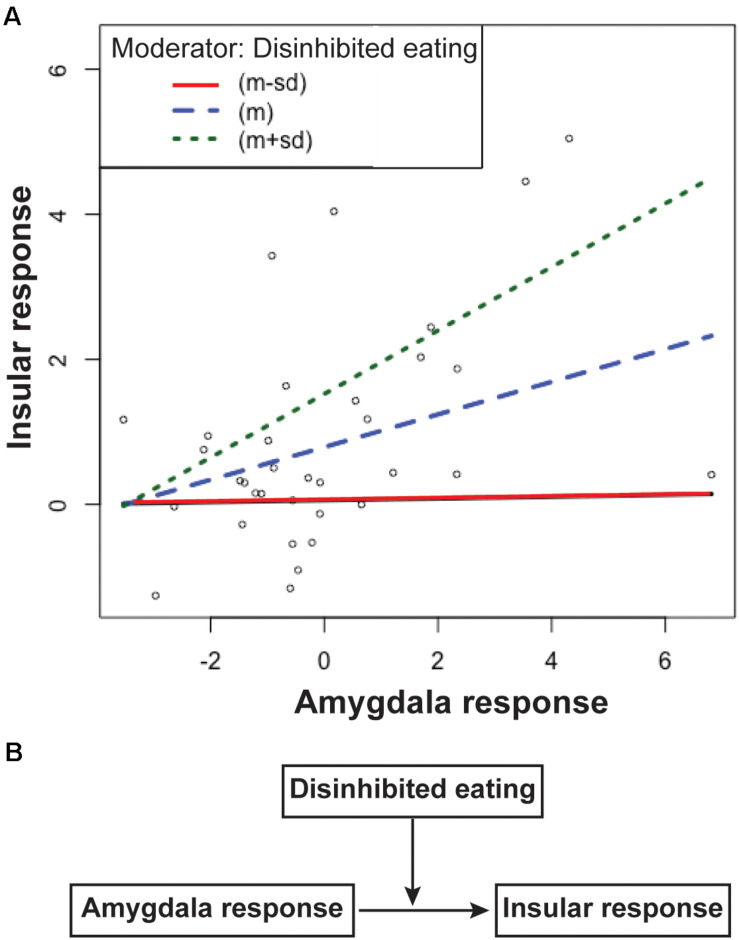
The simple slopes of the disinhibited eating effect **(A)** and the scheme of this moderation model **(B)**. Data are presented as one standard deviation above and one standard deviation below the mean. Participants with less disinhibited eating (the red line) show a greater insular response with a greater amygdala response, but a lower insular response than the mean (the blue line). Participants with greater amygdala responses with greater disinhibited eating show a greater insular response (the green line) than the mean. The difference in the slopes between participants with more or less disinhibited eating shows that disinhibited eating moderates the relationship between the insular response and the amygdala response **(A)**. Disinhibited eating modulated the relationship between insular response and amygdala response **(B)**. The *y*-axis depicts beta values extracted from the cluster related to disinhibited eating in the insular cortex. The *x*-axis depicts beta values extracted from the cluster related to motor impulsiveness in the amygdala.

The first regression analysis showed that the insular response related to disinhibited eating was positively associated with the amygdala response related to motor or total impulsivity (estimates of the beta coefficients [b] = 0.25, standard error [SE] = 0.96, *p* = 0.015; *b* = 0.25, *SE* = 0.95, *p* = 0.01, respectively), while disinhibited eating was positively associated with the insular response (*b* = 0.258, *SE* = 0.065, *p* < 0.001; *b* = 0.253, *SE* = 0.062, *p* < 0.001, respectively). The second regression analysis showed that the interaction term between the amygdala response related to motor impulsivity and disinhibited eating was explained a significant increase in variance in the disinhibited eating-related response in the insula (*b* = 0.067, *SE* = 0.027, *p* = 0.02). The interaction term between the amygdala response related to total impulsivity and disinhibited eating was also explained by a significant increase in variance in the disinhibited eating-related response in the insula (*b* = 0.056, *SE* = 0.025, *p* = 0.03). Thus, disinhibited eating was a moderator of the relationship between the insular response related to disinhibited eating and the amygdala response related to impulsivity (Figure 3).

Motor impulsiveness and total impulsiveness show no effect of moderator for the relationship between the insular response and the amygdala response (*p* = 0.51 and *p* = 0.26, respectively).

### Associations Between Insular-Related Functional Connectivity and Disinhibited Eating or Impulsivity

The insular-related functional connectivity map included the amygdala, hippocampus, orbitofrontal cortex, and ventromedial prefrontal cortex (Supplementary Table 3).

**FIGURE 4:**
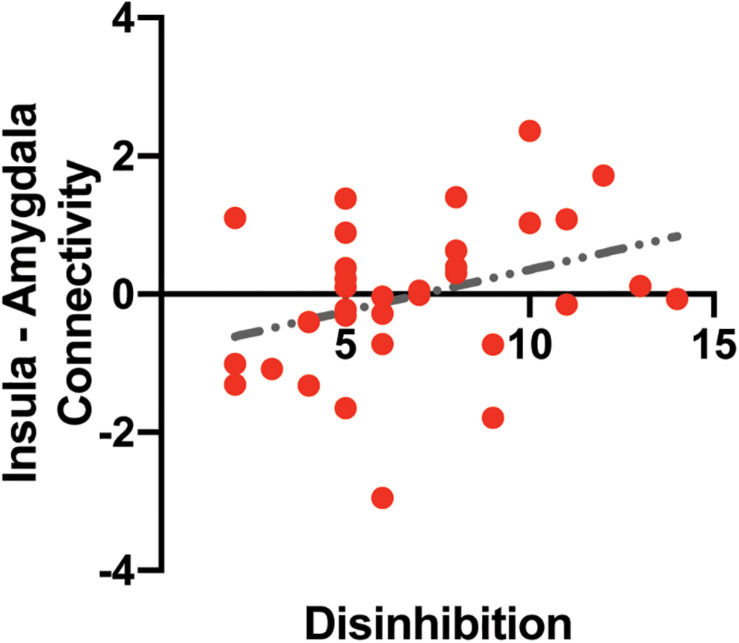
The association between intrinsic insula – amygdala functional connectivity and disinhibited eating. The *y*-axis depicts resting-state insula – amygdala functional connectivity values in *z*-value. The *x*-axis depicts disinhibited eating measured by the disinhibition subscale of the Three-Factor Eating Questionnaire.

A multiple linear regression analysis was used to predict functional connectivity between the insular and the amygdala based on disinhibited eating and sex. Disinhibited eating was positively correlated with functional connectivity between the left amygdala and the insular determined by the fMRI analysis (*b* = 0.146, *SE* = 0.068, *t* = 2.15, *p* < 0.040) (Figure 4). Sex was not associated with functional connectivity (*b* = 0.328, *SE* = 0.425, *t* = 0.77, *p* = 0.446).

A multiple linear regression analysis was also used to predict functional connectivity based on impulsivity and age. Impulsivity (*p* > 0.87) and age (*p* > 0.54) were not associated with functional connectivity.

## Discussion

The present study revealed that greater disinhibited eating was related to an increased insular response to palatable liquid consumption in adolescents although this association was not significant after multiple testing correction, while impulsivity was positively correlated with a greater amygdala response. Moderation analysis also showed that the association between the insula and the amygdala responses was strengthened by the effect of disinhibited eating. Furthermore, greater intrinsic connectivity between the insular and the amygdala was correlated with increased disinhibited eating. Overall, these findings suggest that heightened disinhibited eating may modulate functional connectivity between the amygdala and insula and, in concert with impulsivity, modulate neural reactivity to palatable flavor solution receipt in adolescents.

Unlike previous reports in adolescents (Gallant et al., 2010; Bishop-Gilyard et al., 2011; Rutters et al., 2011; Hootman et al., 2018; Lawless et al., 2020), disinhibited eating was not associated with BMI in this study. There are various assessments of disinhibited eating, such as children’s and parents’ reports on questionnaires, semi-structured interviews, and observations of participants’ eating behavior, and the most thorough assessment would include reliance on as many sources and methods as possible (Shomaker et al., 2011). Therefore, using only one self-report questionnaire could not reflect disinhibited eating well, and thus, there was no significant association between disinhibited eating and BMI in this study. In addition, the BMI in our sample was relatively low (BMI = 20.78 2.05) compared to previous studies (BMI = 21.5–37.5). Given that diet-induced obesity can lead to marked and enduring changes in cognitive control and prefrontal cortex functionality, which, in turn, drives the maintenance of unhealthy eating behaviors (Lowe et al., 2019), increased BMI would strengthen the association between disinhibited eating and BMI. Therefore, the relatively lower BMI in our sample could be another reason why disinhibited eating was not significantly associated with BMI in this study.

Increased impulsivity was weakly associated with greater BMI, in line with previous findings (Cortese et al., 2008; Kalarchian and Marcus, 2012; Schag et al., 2013; Liang et al., 2014; Pearson et al., 2014; Quesada et al., 2018; Kemps et al., 2020). There are several measurements of impulsivity, such as self-report questionnaires and behavioral or cognitive tasks, and compared to questionnaire measures of impulsivity, behavioral task measures of impulsivity are better predictors to estimate the association between impulsivity and BMI (Emery and Levine, 2017). Previous studies with a significant association between impulsivity and BMI have used a behavioral task to measure impulsivity (Fields et al., 2013) or a large sample size (*n* = 51043) (Bénard et al., 2017). Therefore, differences in measurements of impulsivity or sample size would explain why impulsivity did not show a significant association with BMI in this study.

In contrast to previous studies in adults (Fields et al., 2013; Goldstein et al., 2014; Legenbauer et al., 2018), disinhibited eating was not significantly correlated with impulsivity in the present study. In accordance with previous findings showing that changes in the dopaminergic system enhance impulsivity in adolescents (Reichelt, 2016; Dai and Scherf, 2019), we found that impulsivity and disinhibited eating were positively associated with age. However, the significance of the effect of age on impulsivity was different from that for disinhibited eating. These findings suggest that the neural circuits underlying impulsivity may differ from those of disinhibited eating and that these neural circuits could be differentially influenced by maturation. Thus, in contrast to adults (Fields et al., 2013; Goldstein et al., 2014; Legenbauer et al., 2018), we did not observe a significant association between impulsivity and disinhibited eating, as previously reported in adolescents (Fields et al., 2013).

Disinhibited eating is associated with brain activation to flavor solution receipt in the reward and motivative brain regions (Aviram-Friedman et al., 2018; Drummen et al., 2019) and with increased regional blood flow in the insula after liquid meal consumption (DelParigi et al., 2005). In line with these findings, we found that increased disinhibited eating was associated with a greater insular response, which is engaged in the reward aspect of taste stimuli (van Rijn et al., 2018). Additionally, the insula is associated with binge eating behavior (Schienle et al., 2009) and increased adiposity (Rothemund et al., 2007). Therefore, greater disinhibited eating could lead to an increased insular response to palatable flavor solution receipt and let adolescents be more prone to overeating in the presence of palatable foods.

Greater impulsivity was associated with an increased amygdala response to palatable liquid consumption. The amygdala is a key region controlling impulsivity (Kim et al., 2018) and an increased amygdala response to food cues is linked to impulsivity (Beaver et al., 2006; Lowe et al., 2009; Kerr et al., 2015), adiposity (Stoeckel et al., 2008; Sun et al., 2015), binge-eating (Schienle et al., 2009), and disinhibition (Dietrich et al., 2016). Thus, increased impulsivity may result in a greater amygdala response to palatable food consumption, which may contribute to maladaptive eating in adolescents.

Although we found no direct association between disinhibited eating and impulsivity, adolescents with increased disinhibited eating showed a greater association between the insula and the amygdala responses to palatable liquid consumption. Additionally, increased disinhibited eating was positively associated with strengthened intrinsic connectivity between the insula and amygdala. Furthermore, as previously reported (Doornweerd et al., 2018; Nakamura et al., 2020), these regions jointly responded to gustatory stimuli, while a response to food cues in these regions is involved in binge-eating (Schienle et al., 2009). Moreover, in line with previous findings, which show that the insula and amygdala were functionally and anatomically connected (Ghaziri et al., 2018; Sylvester et al., 2020), the seed-based functional connectivity analysis revealed that the intrinsic functional connectivity map of the insula included the regions related to disinhibited eating and impulsivity, such as the amygdala and orbitofrontal cortex (Beaver et al., 2006; Lowe et al., 2009; Maayan et al., 2011; Kerr et al., 2015; Dietrich et al., 2016; Zhao et al., 2017). This result indicates that the insular response related to disinhibited eating is connected to the intrinsic neural circuit involved in disinhibited eating and impulsivity. Altogether, although we did not find a significant direct association between disinhibited eating and impulsivity, these behavioral characteristics may jointly modulate neural circuits of ingestive behavior and drive adolescents to maladaptive eating.

There are several limitations in the present study that could be addressed in future research. First, the sample size in this study was small (*n* = 34). Although the *post hoc* statistical power analysis of the flavor stimulus fMRI data analysis showed that the statistical power was acceptable, it is favorable that future studies include more participants. Second, we examined the associations across disinhibited eating, impulsivity, and functional connectivity. However, causal relationships across these variables should be examined in future longitudinal studies. Third, although we only included teenagers (14–19 years old), the associations between disinhibited eating or impulsivity and neural responses to palatable liquid consumption may differ over this age range because reward, impulsivity, and disinhibition control regions are dramatically modified during adolescence (Spear, 2013; Smith et al., 2014; Dai and Scherf, 2019). Future studies should examine the effect of impulsivity and disinhibited eating on neural circuits of ingestive behavior using adolescents with a smaller age range. Fourth, we measured impulsivity using the BIS-11. There are several ways to measure impulsivity and each measurement assesses slightly different dimensions of impulsivity, which are differentially associated with adiposity (Emery and Levine, 2017). Thus, future studies should use different measurements to test the associations between different dimensions of impulsivity and disinhibition or neural circuits. Fifth, in this study, five different beverages were used as flavor stimuli. To measure the brain response to rewarding food stimuli, each individual’s preferred flavor solution was selected from the five options. Future studies should be performed to control for differences in the sensory properties of solutions, given that these differences in the beverage could influence the brain response to flavor stimuli.

## Conclusion

Overall, both disinhibited eating and impulsivity were related to a greater response to palatable liquid consumption in the gustatory and reward regions, which are associated with binge-eating. Adolescents with increased disinhibited eating also showed greater associations between the insula and amygdala responses to palatable liquid consumption, while increased disinhibited eating was positively associated with strengthened intrinsic connectivity between the insula and the amygdala. Therefore, heightened disinhibited eating may modulate functional connectivity between the amygdala and the insula and, in concert with impulsivity, modulate neural reactivity to palatable flavor solution receipt and drive adolescents to maladaptive eating. Given these findings, disinhibited eating may be an important target for successful interventions for maladaptive eating in adolescents.

## Data Availability Statement

The original contributions presented in the study are included in the article/Supplementary Material, further inquiries can be directed to the corresponding author/s.

## Ethics Statement

The studies involving human participants were reviewed and approved by The Ethics Committee of the Department of Arts and Sciences, The University of Tokyo. Written informed consent to participate in this study was provided by the participants’ legal guardian/next of kin.

## Author Contributions

YN and SK: conceptualization and design and reviewing and editing the manuscript. YN: acquiring the data. YN: analyzing the data and writing the manuscript. Both authors contributed to the article and approved the submitted version.

## Conflict of Interest

The authors declare that the research was conducted in the absence of any commercial or financial relationships that could be construed as a potential conflict of interest.

## Footnotes

1 http://www.R-project.org/

## Supplementary Material

Supplementary Table 1 Ratings for internal state.

Supplementary Table 2 Brain response to [flavored solution > tasteless solution].

Supplementary Table 3 Functional connectivity related to the cluster in the insular cortex.
