# Supplementary material for: Association of Disinhibited Eating and Trait of Impulsivity With Insula and Amygdala Responses to Palatable Liquid Consumption
Source: Front Syst Neurosci. 2021 May 3;15:647143. doi: 10.3389/fnsys.2021.647143 (PMC8128107; doi:10.3389/fnsys.2021.647143)
Supplement: Supplementary Table 1 — Ratings for internal state. [file Table_1.docx]

**Supplementary Material**

**Supplementary Table 1** **|** Ratings for internal state.

|  | **Hunger at the flavor stimulus scan** | **Hunger at the resting state scan** | **Fullness at the flavor stimulus scan** | **Fullness at the resting state scan** |
| --- | --- | --- | --- | --- |
| Mean | 4.12 | 4.47 | 4.91 | 4.47 |
| S.D. | 1.12 | 1.26 | 1.16 | 1.24 |

*S.D., standard deviation.*

**Supplementary Table 2** **|** Brain response to [flavored solution > tasteless solution].

| **Brain region** | **Side** | **MNI coordinate** | | | **Cluster size** | ***z*-value** | ***p*_FWE-corrected_ value** |
| --- | --- | --- | --- | --- | --- | --- | --- |
|  |  | **x** | **y** | **z** |  |  |  |
| Insula | R | 42 | −10 | 2 | 79 | 4.15 | 0.026 |
|  | L | −36 | −8 | −10 | 18 | 4.03 | 0.039 |
| Amygdala | R | 30 | −2 | −26 | 67 | 4.41 | 0.001* |
|  | L | −22 | −4 | −20 | 139 | 4.59 | 0.001* |
| Hippocampus | R | 26 | −4 | −26 | 157 | 4.29 | 0.008 |
|  | L | −22 | −10 | −24 | 299 | 5.01 | < 0.001* |
| Parahippocampus | R | 22 | −10 | −24 | 160 | 4.27 | 0.01 |
|  | L | −24 | −12 | −26 | 85 | 4.81 | 0.001* |
| Striatum (putamen) | L | −30 | −12 | −8 | 12 | 4.07 | 0.038 |
| Thalamus (lateral thalamic nuclei) | L | −8 | −6 | −2 | 5 | 3.79 | 0.048 |
| Pons | L | −2 | −30 | −36 | 19 | 3.84 | 0.041 |

*Significant threshold was set at *p_FWE-corrected_* < 0.003 (0.05/14).

**Supplementary Table 3** **|** Functional connectivity related to the cluster in the insular cortex.

| **Brain region** | **Side** | **MNI coordinate** | | | **Cluster size** | ***z*-value** | ***p*_FWE-corrected_ value** |
| --- | --- | --- | --- | --- | --- | --- | --- |
|  |  | **x** | **y** | **z** |  |  |  |
| Hippocampus - amygdala | Bilateral | −28 | −14 | −14 | 65204 | 8.29 | < 0.001 |
| Orbitofrontal cortex | L | −38 | 32 | −8 | 545 | 4.59 | < 0.001 |
